# A Review of the Combination Therapy of Low Frequency Ultrasound with Antibiotics

**DOI:** 10.1155/2017/2317846

**Published:** 2017-10-16

**Authors:** Yun Cai, Jin Wang, Xu Liu, Rui Wang, Lei Xia

**Affiliations:** Center of Medicine Clinical Research, Department of Pharmacy, PLA General Hospital, Beijing, China

## Abstract

Single antimicrobial therapy has been unable to resist the global spread of bacterial resistance. Literatures of available* in vitro* and* in vivo* studies were reviewed and the results showed that low frequency ultrasound (LFU) has a promising synergistic bactericidal effect with antibiotics against both planktonic and biofilm bacteria. It also can facilitate the release of antibiotics from medical implants. As a noninvasive and targeted therapy, LFU has great potential in treating bacterial infections. However, more in-depth and detailed studies are still needed before LFU is officially applied as a combination therapy in the field of anti-infective treatment.

## 1. Introduction

After the application of the first antibiotic penicillin, a series of natural, semisynthetic, and synthetic antimicrobials were discovered and applied in clinics, achieving great progress in bacterial infection therapy and saving millions of lives at the golden age of antibiotics [1]. However, many decades later, bacterial infections have again become a serious threat due to lack of new drug development and rapid emergence of resistant bacteria. Facing the global “antibiotic resistance crisis,” we are now in the “postantibiotic era” [2]. For example, tigecycline and colistin are the last-resort antibiotics for multidrug resistant (MDR)* Acinetobacter*, which is defined as a serious threat to human health [3]. Unfortunately, tigecycline-resistant* Acinetobacter baumannii *was reported only 2 years after tigecycline was approved by FDA in 2005 [4]. Colistin heteroresistant and resistant* A. baumannii* have also been described worldwide [5]. In this context, antimicrobial combination therapy has become an option to treat infection with MDR bacteria because of broad coverage and synergistic effect. However, it also brings higher risk of adverse events, leading to treatment failure, increased antibiotic use, and possible accelerated emergence of drug resistance [6].

Overall, we need other methods to confront the growing problem of bacterial resistance. Low frequency ultrasound (LFU) is one of the safe and promising physical methods [7]. Ultrasound, a pressure sound wave with frequency of 20 kHz or more, has been used for decades in research and diagnostics. LFU generally has a frequency ranging from 20 to 100 kHz and is also termed as high-power ultrasound [8]. It is believed that the acoustic cavitation, or the growth and collapse of microbubbles in liquid media, is the underlying mechanism for the bactericidal effects of ultrasound because it could generate mechanical forces such as shock waves, shear forces, and microjets to damage microorganisms [9]. The stable cavitation and radiation pressure will generate multidirectional acoustic microstreams, which will produce a high shear stress to enhance the release and delivery of antibiotic from imbedded implants [10]. With other advantages such as beam directivity and capability of treating deep tissue targets without tissue damage, LFU has been reported in series of studies to be a promising method to enhance the antibiotic action on bacteria. Based on available* in vitro* and* in vivo* data, we aimed to evaluate the synergistic effects of LFU and antibiotics combination therapy in future clinical practice in this review.

## 2. Synergistic Effects of LFU and Antibiotics against Planktonic Bacteria

**Figure 1:**
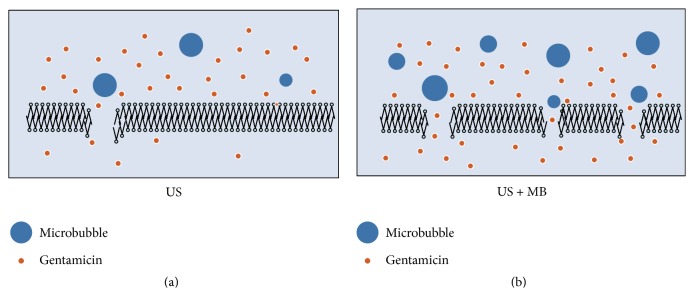
Nonthermal effect of stable cavitation by microbubble-mediated ultrasound. The mechanical effect of cavitating bubbles created pores in the cell membrane. This allowed gentamicin to enter the bacteria via passive diffusion. (a) In ultrasound condition, there were sparse microbubbles and only a few gentamicin particles passed through cell membrane. (b) Addition of external microbubbles strengthened the cavitation, created more pores, and drove more drugs through bacterial cell membrane [16].

Although many studies showed that LFU alone can significantly reduce bacterial counts [8, 11–13], Pitt et al. [14] first confirmed the synergistic effects of LFU and antibiotics in 1994 (Table 1). They evaluated the combination of LFU and gentamicin against planktonic cultures of* Pseudomonas aeruginosa (P. aeruginosa), Escherichia coli (E. coli), Staphylococcus epidermidis (S. epidermidis)*, and* Staphylococcus aureus (S. aureus)* and found that, at the level of 67 kHz and 0.3 W/cm^2^ intensity, continuous ultrasound alone had no bacteria inhibitory or bactericidal activity, but, in combination with the LFU, the minimum inhibitory concentrations (MICs) of gentamicin for* P. aeruginosa* and* E. coli *were reduced from 4 to 3 *μ*g/ml and 6 to 3 *μ*g/ml, respectively, and the viability of bacteria decreased by several orders of magnitude. However, ultrasonic treatment enhanced activity of antibiotics was not observed with Gram-positive* S. epidermidis* and* S. aureus*. Williams and Pitt [15] also observed the combined effect of gentamicin and LFU against* E. coli*. The greatest bactericidal effect (approximately 5 log reductions in viable population) was realized with continuous ultrasonic insonation at 70 kHz and 4.5 W/cm^2^. Zhu et al. [16] confirmed that microbubble-mediated LFU could further enhance the antimicrobial efficacy of gentamicin compared with LFU alone. These might be because the addition of external microbubbles strengthened the cavitation, created more pores, and drove more drugs through bacterial cell membrane (Figure 1). Rapoport et al. [17] applied a spin-labeled gentamicin bioreduction kinetics model to reveal the mechanism of this synergism against Gram-negative* P. aeruginosa* and* E. coli*. Hydrophilic gentamicin is assumed to function through porin channels in the outer cell membranes. The results showed the penetration of spin-labeled gentamicin was not affected by continuous insonation with intensity below the cavitation threshold (2.4 W/cm^2^), implying that the synergistic effect between hydrophilic antibiotics and LFU in killing Gram-negative bacteria did not result from the enhanced antibiotic penetration through bacterial cell walls. They speculated that it might be caused by the effect of ultrasound on interaction of antibiotics with bacterial cells. It has been reported that although* P. aeruginosa* is resistant to erythromycin (MIC between 350 and 250 *μ*g/ml), combination of erythromycin with continuous LFU at 70 kHz and 2.2 W/cm^2^ LFU enhanced the bactericidal effect of 125 *μ*g/ml erythromycin against* P. aeruginosa* [18]. Rapoport et al. [19] found that application of continuous ultrasound to suspended* P. aeruginosa* cells resulted in increased uptake of hydrophobic antibiotic erythromycin. This effect was different from that on hydrophilic antibiotics because the penetration of lipophilic compounds proceeds through the phospholipid bilayers rather than the porin channels. The data suggested that, in contrast to porin channels, phospholipid bilayers are perturbed by insonation, forming some transient defects responsible for the enhanced penetration of lipid-soluble substances. The effect was transient because the initial membrane permeability was restored after the termination of insonation. Runyan et al. [20] found that the rate of nitrocefin hydrolysis, which reflects the entry rate of antibiotics, in suspension of* P. aeruginosa* was increased by application of continuous ultrasound in an intensity-dependent manner. Liu et al. [21] evaluated the antibacterial effect of fluoroquinolones (levofloxacin and ciprofloxacin) on* E. coli* with and without stimulation of continuous ultrasound at 40 kHz and found that addition of LFU enhanced the killing rate of fluoroquinolone to* E. coli* by 10–30% compared with fluoroquinolone alone. Moreover, they found that ^*∙*^O^2−^ and ^*∙*^OH produced through combined effect of LFU and fluoroquinolones might be the main reason for the enhanced bactericidal effect. Rediske et al. [22] compared the action of LFU combined with antibiotics on several bacterial species. They found that gentamicin, kanamycin, or streptomycin at MIC level in combination with continuous ultrasound decreased viability of* E. aerogenes* by 4-, 2-, and 1-log degree than exposure to antibiotic alone for 3 h. The killing rate of gentamicin at the MIC in combination with ultrasound on* S. marcescens* and* S. derby* was 2-3 logs greater than that with antibiotic alone.

Although Pitt et al. [14] showed that LFU alone did not enhance gentamicin activity against Gram-positive strains, combination of LFU with other antibiotics showed synergistic bactericidal effect on* S. aureus *and* S. epidermidis* (Table 1). Ayan et al. [23] investigated the effects of eight antibiotics, namely, penicillin, oxacillin, erythromycin, teicoplanin, vancomycin, clindamycin, levofloxacin, and ciprofloxacin, in combination with LFU, and found combined treatment of antibiotics with continuous LFU at 1.5 MHz and 30–161 mW/cm^2^ significantly lowered the number of bacterial colonies compared with that of treatment with antibiotics alone (*P* < 0.001). Conner-Kerr et al. [24] conducted an* in vitro* study to determine the effects of continuous LFU delivered at 35 kHz and 2 W/cm^2^ on bacterial viability, cell wall structure, and colony characteristics, including antibiotic resistance on Methicillin-resistant* S. aureus* (MRSA). They found that combined treatment of LFU increased the inhibition zone of 1 *μ*g oxacillin on MRSA plates to above 13 mm, the critical value for determining antibiotic resistance, indicating that combined treatment of LFU decreased bacterial resistance to oxacillin. Rediske et al. [22] reported that bactericidal effect of ampicillin in combination with continuous ultrasound at 70 kHz and 3 W/cm^2^ against* S. epidermidis* was 1.5 logs greater than that of ampicillin alone.

At present, only* in vitro* experiments were applied against planktonic bacteria. Using* in vitro* experiments can easily determine the antibacterial effect and quickly suggest whether the combination of LFU and specific antimicrobial agents has a synergistic antibacterial effect on a certain bacterium. Most of these studies applied continuous ultrasound with frequency ranging from 35 to 70 Hz and intensity lower than 4.7 W/cm^2^. Only one study applied higher frequency (1.5 MHz) and also showed the synergistic bactericidal effect with antibiotics. Although these results are promising, differences in the critical ultrasound parameters among these studies are evident. Especially for ultrasound time, some studies showed obvious synergism after exposure to LFU within 30 min, while others apply continuous ultrasound for 24 h or even longer.

## 3. Synergistic Effects of LFU and Antibiotics against Biofilm

Biofilms are microbes attached to surfaces or to each other in aggregates or clumps. Biofilms show extreme tolerance to antimicrobials and host defenses and can withstand 100–1000 times higher antimicrobial concentrations than planktonic counterparts [25]. Therefore, biofilm caused infection has always been a troubling clinical problem.

Reports of synergistic effects of LFU and antibiotics against biofilm were summarized in Table 2. Qian et al. [26] conducted a series of experiments to test the synergism effect of LFU and gentamicin on biofilm of* P. aeruginosa*. First, they found that continuous LFU at 500 kHz and 10 mW/cm^2^ enhanced the bactericidal effect of gentamicin against 24 h old biofilm. In addition, confocal scanning laser microscopy (CSLM) showed that LFU at 10 mW/cm^2^ did not disrupt biofilm. Therefore, they hypothesized that there is minimal concern that ultrasonic treatment of an implant infection would break up and disseminate clusters of biomass to other parts of the body. Then they compared the effect of ultrasound at different frequencies of 70 kHz, 500 kHz, 2.25 MHz, and 10 MHz, respectively, and at power density of 10 mW/cm^2^. The results indicated lower frequency ultrasound was significantly more effective than higher frequency ultrasound in reducing bacterial viability within the biofilm [27]. In another study they found that continuous waveform ultrasound at frequency of 70 kHz and power density of 10 mW/cm^2^ was more effective in enhancing antibiotic bactericidal effect than that at intensity of 1 mW/cm^2^ and pulsed waveform ultrasound with a burst power density appeared to be as effective as continuous ultrasound at the same power density [28]. Based on these studies, Qian et al. [28] conducted a comprehensive analysis about the possible mechanisms in light of the observed influence of various ultrasonic parameters on the enhanced action of gentamicin against biofilms. First they rejected the hypothesis of oscillatory shear inducing antibiotic uptake. Because mathematical analysis of oscillatory shear stress on the cell shows that the magnitude of stress increases with frequency, the bactericidal effect of ultrasound decreases with its frequency. They also ruled out the existence of transient cavitation in the bioacoustic effect. There is no difference between the viability of the control biofilm and the biofilm exposed to ultrasound only. This indicates that the cells are not killed by the free radicals or extreme environment produced by transient cavitation and the observed bioacoustic effect is related to the temporal peak intensity, not the temporal average intensity of ultrasound. If transient cavitation were involved, the bactericidal effect would be a function of temporal average intensity, not peak intensity. Finally, because the dependence upon peak power density suggests that acoustic pressure plays a significant role, they speculated it is possible that stable cavitation and the accompanying microstreaming contribute to the bioacoustic effect.

Some* in vitro* and* in vivo* experiments aimed at biofilm of* E. coli *also showed enhanced efficacy of gentamicin by LFU. An* in vitro* study found that, after 6 h of combined gentamicin and ultrasound treatment, a single-species* E. coli* biofilm showed no reproductive ability. The combination of continuous wave ultrasound at 70 kHz with gentamicin significantly reduced bacterial viability compared with antibiotic alone and resulted in about 97% killing within 2 h. When the exposure time was extended to 6 h, the combination of ultrasound and gentamicin completely inhibited the reproductive ability of the biofilm. However, the combination of 500 kHz ultrasound and antibiotic produced only a slight reduction [29]. Rediske et al. [30] developed an* in vivo* rabbit model with biofilm-infected disks implanted to determine if continuous ultrasound at 28.48 kHz could enhance the effects of gentamicin. They found that treatment alone with ultrasound at 100 mW/cm^2^ did not affect the viable counts of bacteria, but treatment with antibiotic plus ultrasound reduced the viable counts of bacteria by 2.39 log⁡10 fold (*P* = 0.048) compared with treatment with antibiotic alone. In addition, treatment with ultrasound at 100 mW/cm^2^ showed no damage to the rabbit skin, but treatment with ultrasound at 300 mW/cm^2^ did. To minimize the skin damage, they evaluated the pulsed ultrasound at 300 or 600 mW/cm^2^ in a pulse of 100 cycles with a 1 : 3 or 1 : 6 duty cycle, wherein the temporal average intensity was 100 mW/cm^2^, and found that ultrasound enhanced the action of gentamicin in killing* E. coli* but did not damage skin at pulse or continuous intensity of at least 300 mW/cm^2^ and an average intensity of 100 mW/cm^2^ [31].

Carmen et al. [32] conducted* in vitro* and* in vivo* researches on LFU combined with gentamicin or vancomycin against* P. aeruginosa* and* E. coli* biofilm. Their* in vitro* study using colony biofilms found that ultrasound at 70 kHz and 1.5 W/cm^2^ can significantly increase gentamicin transport through biofilms and 45 min of insonation can double the amount of gentamicin in* E. coli* biofilm compared to their noninsonated counterparts. In addition, no detectable gentamicin penetrated* P. aeruginosa* biofilms without ultrasound. But more than 0.45 mg gentamicin was collected after 45 min of insonation. Their* in vivo *study on rabbits implanted subcutaneously with infected biofilm disks showed that application of pulsed ultrasound in a 1 : 3 duty cycle at 28.5 kHz and 500 mW/cm^2^ with 48 h of gentamicin treatment resulted in a significant reduction of 2.28 ± 0.83  log 10 CFU/cm^2^ in* E. coli* biofilm. However, 24 or 48 h of ultrasound combined with gentamicin failed to significantly enhance the killing of* P. aeruginosa* in the biofilm. This difference may be related to the documented difference in outer membrane permeability between* P. aeruginosa* and* E. coli* [33]. In another study, they found that* S. epidermidis* biofilms responded favorably to combinations of ultrasound and vancomycin, because 48 h of ultrasound significantly reduced viable bacteria in the biofilm by 2.08 log 10 CFU/cm^2^ [34].

Seth et al. [35] used a rabbit* in vivo* wound biofilm model. They topically applied ciprofloxacin on postoperative day 4 to eliminate planktonic* P. aeruginosa* and an antimicrobial absorbent dressing containing polyhexamethylene biguanide to prevent regrowth of planktonic bacteria and carried out LFU treatment every other day or every day. They found that applications of LFU significantly impacted biofilm-infected wounds, including a decrease in viable bacteria, as well as an overall improvement in wound healing and host inflammatory dynamics. Human *β*-defensin 3 (HBD-3) is a promising cationic antimicrobial peptide for future bactericidal employment. One study showed that biofilm density, the percentage of live cells, and the viable count of* Staphylococcus* that recovered from the biofilm on the titanium surface in mice were significantly decreased after combined treatment of HBD-3 with ultrasound. Moreover, ultrasound could enhance HBD-3 activity of inhibiting the biofilm-associated genes expression [36]. Liu et al. [37] found that although application of LFU (40 kHz, 600 mW/cm^2^, 30 min, duty cycle 1 : 9) alone or in combination with single antibiotic (colistin or vancomycin) failed to significantly reduce bacteria counts in* A. baumannii* biofilms, application of LFU in combination with both colistin and vancomycin apparently had more effective antibacterial function against biofilm.

Both the* in vitro* and animal model studies have been applied in the LFU combinations studies against biofilm. In addition to confirming that LFU has a synergistic antimicrobial effect on biofilms, these series studies further clarified three key issues. First, at the same intensity, lower frequency ultrasound is more effective than higher frequency ultrasound in reducing bacterial viability within the biofilm. Secondly, synergistic antimicrobial effect of pulsed-wave ultrasound is related to the temporal peak intensity, not the temporal average intensity of ultrasound. Thirdly, skin damage is related to the average intensity of ultrasound. The higher the average ultrasound intensity, the greater the damage to the skin. These suggest that maximum sterilization and minimized damage to skin can be achieved by adjusting the duty cycle of pulsed LFU. From the* in vivo* data included in these reviews, although the study design was different, LFU at 20–30 kHz with the intensity range from 200 to 500 mW/cm^2^ seems to have most successful outcomes in enhancing bactericidal effect against biofilm.

## 4. Effect of LFU on Antibiotic Release from Implanted Material

The high incidence of device-related biofilm infections has spurred a rapidly growing field of research directed at controlling or eliminating biofilm formation. Various device-related infections have been well documented on vascular catheters as well as prosthetic hips, knees, and other orthopedic implants [38]. In recent years great efforts have been devoted to create biocompatible materials that prevent or minimize biofilm infection by inhibiting the formation and survival of biofilms. One strategy is to incorporate antibiotics into the devices or materials, which targets the site where biofilm formation is likely to occur. LFU plays an important role in facilitating antibiotic release. For example, Norris et al. [39] assessed the efficacy of ultrasonically controlled release of ciprofloxacin from self-assembled coatings on poly(2-hydroxyethyl methacrylate) hydrogels against* P. aeruginosa *biofilm and showed application of LFU enhanced release of ciprofloxacin, which was retained inside the polymer in the absence of ultrasound. And biofilm accumulation on ciprofloxacin-loaded hydrogels with ultrasound-induced drug delivery was significantly reduced compared to that in the control experiments (Table 3).

The most frequently reported research on LFU promotes the release of antibiotics was about bone cement (Table 3). Hendriks et al. [40] first compared the response of three commercially available bone cements to ultrasound and found a striking increased release of gentamicin upon ultrasound at 46.5 kHz and 167 mW/cm^2^. They also carried a series experiment to test the effects of LFU on gentamicin-loaded beads or bone cements by measuring gentamicin release from both materials after 18 h of exposure in PBS and found that ultrasound significantly increased gentamicin release from beads, but only marginally from bone cements [41]. Then they investigated the effect of gentamicin released from bone cements treated with LFU on 4 strains derived from patients, including* E. coli, S. aureus, *coagulase-negative staphylococci, and* P. aeruginosa. *They found that ultrasound plus gentamicin further reduced bacterial viability of both planktonic and biofilm bacteria and the percentage of reduction was higher for biofilm bacteria than for planktonic bacteria. They speculated that this was probably due to the slow diffusion of the released antibiotic through the biofilm in the suspension, causing a higher local concentration of antibiotic in the biofilm than in suspension [42]. Moreover, LFU in combination with different administration of gentamicin on bone cements was assessed in rabbit model. Compared to the negative controls, ultrasound resulted in more than 50% enhancement in bacterial killing in combination with both systemic gentamicin and gentamicin released from antibiotic-loaded bone cement [43].

**Figure 2:**
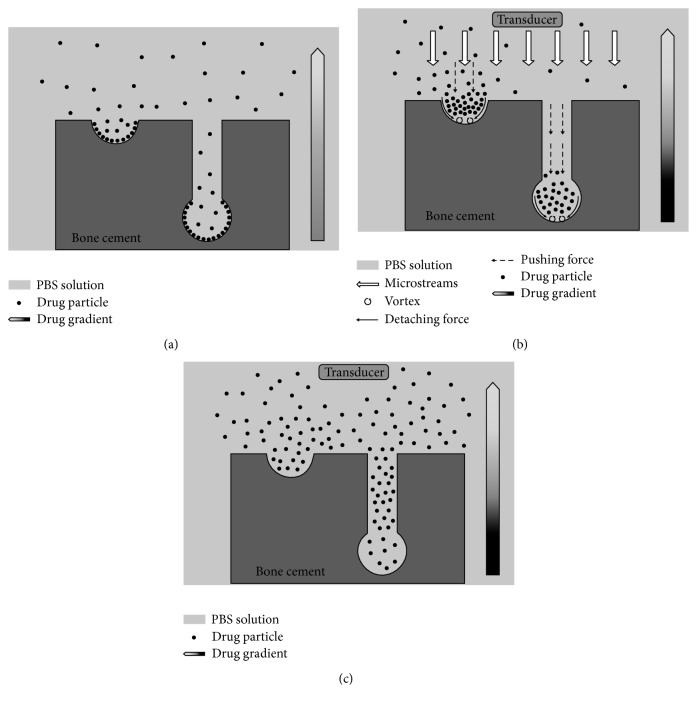
Possible mechanisms for the improvement of intermittent watt-level ultrasonication on vancomycin release from acrylic cement. (a) No ultrasonication. A large number of drug grains resided in the craters and the bottoms of pores through adhering to cement matrix, only a little fraction accessed the external PBS. (b) Ultrasonication is on, and the detaching force by microstreams produced vortex at the drug-cement interface during the ultrasonication period. Large quantities of drug grains were desorbed. However, the pushing force, another force by microstreams, hampered the drug from outward diffusion through the channels or craters into the external PBS. (c) Pushing force disappeared in the pause period for intermittent ultrasonication. The desorbed grains diffuse readily out of the craters and pores through the concentration gradient, which was built up during the ultrasonication period [10].

Cai et al. [44] investigated the effect of pulsed-wave LFU on the antimicrobial efficacy of vancomycin on acrylic bone cement. After implanting cement and inoculating* S. aureus* into the bilateral hips of rabbits, ultrasound was applied to animals in the normal ultrasound group at 0–12 h of postoperation and to those in the delayed-ultrasound group at 12–24 h of postoperation. The results showed that the length of time when local drug level exceeded the minimum inhibitory concentration (*T* > MIC) was significantly prolonged in the delayed-ultrasound group compared with that in the ultrasound untreated or normal ultrasound groups. In addition, bacterial densities in both right hip aspirates and right femoral tissues at 48 h reduced the most in the delayed-ultrasound group. They also revealed that intermittent ultrasonication (a 10 min pause between two 40-min ultrasonic periods) improved vancomycin release from cement in view of prolonged *T* > MIC compared with continuous ultrasonication [10]. The mechanisms involved in the ultrasound-enhanced drug release from cement were attributed most to the nonthermal effect of ultrasound, mainly the stable cavitation and the radiation pressure, which generate multidirectional acoustic microstreams. The microstream produced a high shear stress at drug-cement interfaces, allowing detachment of drug grains from the surface. Meanwhile, the microstream pushed solution into acrylic matrix via craters and channels (Figure 2) [10]. They further investigated whether microbubbles-mediated ultrasound could facilitate vancomycin elution from cylindrical specimens and enhance activity of the eluted antibiotic against* S. aureus*. The* in vitro* and* in vivo* results all showed that both elution and activity of vancomycin were significantly higher in vancomycin + microbubbles + ultrasound specimen than in vancomycin or vancomycin + ultrasound specimens [45].

Yan et al. [46] investigated the enhancement of continuous ultrasound on vancomycin release and antimicrobial efficacy of antibiotic on acrylic bone cement. The results showed that ultrasound increased the drug elution by 2.57–27.44% when compared with the controls* in vitro*. Vancomycin concentrations in the rabbit hip cavity and urinary elimination of vancomycin were both measured after exposure to ultrasound. The results showed that continuous ultrasound increased local *T*_max_ by 47.6 mg/mL and urinary elimination of vancomycin by 109.56 mg but failed to prolong the local *T* > MIC. Wendling et al. [47] developed a study to determine the effect of different mixing techniques of vancomycin-impregnated polymethylmethacrylate cement with LFU on antibiotic elution. They found that the combination of a delayed mix technique with LFU treatments could significantly increase both short- and long-term antibiotic elution without affecting mechanical strength.

Pulse ultrasound is most commonly applied in these studies. Some studies have evaluated the synergistic antimicrobial effects of LFU using pharmacokinetics/pharmacodynamics and demonstrate the ability of LFU in promoting drug release in bone cement. However, in addition to the frequency, many other factors also affect the release of antibiotics, such as intensity and the treatment duration time of LFU itself, bone cement type, mixing technique, and the beginning ultrasound time after implantation. At the same time, only a few studies examined the effect of ultrasound on the physical properties of the implant. According to the current limited results, it can only be roughly speculated that 20–50 kHz and 100–300 mW/cm^2^ are relatively effective frequency and intensity range of LFU in promoting antibiotic release from implanted materials. However, more comprehensive and in-depth research is still needed.

## 5. Clinical Practice of LFU and Antibiotic Combination Therapy

As a therapeutic adjuvant, LFU has been extensively studied in chronic wound healing and offers relatively painless debridement and bacterial biofilm destruction. For example, Breuing et al. [48] explored the effect of LFU on wounds in 17 patients and found that wound was healed in 9 (53%) patients with or without the aid of skin grafts and reduced by at least 50% in size in 6 (35%) and by 20%–30% in 2 patients. In addition, no patients required initiation of antibiotic treatment after starting LFU. Tewarie et al. [49] compared the ultrasonic debridement (*n* = 18) with conventional surgical therapy (*n* = 19) in removing bacterial biofilms and preservation of vital sternal tissue in 37 consecutive patients. Time to secondary wound closure following eradication was significantly shortened in LFU group. Postoperative antibiotic treatment time and recurrence of sternocutaneous fistula also showed a trend in favor of LFU group. However, only few clinical reports exist on the combined application of LFU and antibiotics. Only Komrakov and Antipov [50] reported that combined use of LFU and gentamycin solution for treatment of wounds in 17 patients reduced the critical level of bacterial wound colonization. They found that this combination could decrease the incidence of purulent-septic complications from 35.7 to 5.9%. There was no wound suppuration in 14 patients after the operations for late reocclusion performed in the presence of cicatrices of the tissues.

## 6. Conclusions

Based on the available* in vitro* and* in vivo* data, it can be concluded that LFU can assist the antibiotic action on both planktonic and biofilm bacteria. For antibiotic imbedded implants, LFU can promote the release of antibiotics to achieve the optimal efficacy. However, there is still a long way to go before clinical application of combination therapy of LFU with antibiotics. First of all, the current studies involved a narrow range of susceptible pathogens. There are very few studies on the most threatening MDR bacteria. Secondly, frequency, intensity, and pulse cycle varied a lot at present. The promising frequency and intensity from* in vitro* studies are likely to cause local damage in* in vivo* studies [30, 31]. Therefore, LFU parameters appropriate for clinical application need to be further explored. Thirdly, one study indicated that LFU treatment reduced the interface shear strength and initial stability of vancomycin-loaded acrylic bone cement-stem [51]. So the impact of LFU on the physical properties of the implant materials requires a comprehensive examination. At last, because bacteria will partially be removed from the biofilm surface when LFU is applied [52], whether it will bring the risk of spreading the pathogens and forming systemic bloodstream infection also requires more careful evaluation.

## Figures and Tables

**Table 1:** Reports about effect of antibiotics combined with LFU on planktonic bacteria.

Authors (year)	Type of research	Pathogens	Frequency, density, and time of LFU	Combined antibiotics	Results
Pitt et al. (1994) [14]	*In vitro* study on planktonic cultures	*P. aeruginosa* *E. coli* *S. epidermidis* *S. aureus*	67 kHz 0.3 W/cm^2^ CW, 1, 2, 3, 6, 9, 12, 14, 18, 24 h	Gentamicin	A synergistic effect was observed and bacterial viability of *P. aeruginosa* and *E. coli* was reduced by several orders of magnitude when gentamicin and ultrasound were combined, while ultrasound alone did not kill bacteria. The synergistic effect was not observed with *S. epidermidis* and *S. aureus*.

Williams and Pitt (1997) [15]	*In vitro* study on planktonic cultures	*E. coli*	70 kHz 0.01–4.5 W/cm^2^ CW, 1, 2, 3 h	Gentamicin	Combined with ultrasonication greatly enhanced the activity of gentamicin. The greatest bactericidal effect (approximately 5-log reduction in viable population) was realized at 4.5 W/cm^2^ and decreased with reductions in power density. At 10 mW/cm^2^, no significant acoustic enhanced bactericidal effect was noted.

Rapoport et al. (1997) [19]	*In vitro* study on planktonic cultures	*P. aeruginosa*	80 kHz 0.68, 3.2 W/cm^2^ CW, 2, 4, 6 h	Erythromycin	The efficiency of erythromycin in killing planktonic *P. aeruginosa* increased more than an order of magnitude upon the simultaneous application of ultrasound. Ultrasound alone does not kill the cells but rather sensitizes the cells to antibiotic action.

Rediske et al. (1998) [22]	*In vitro* study on planktonic cultures	*E. aerogenes* *S. marcescens* *Salmonella derby* *Streptococcus mitis* *S. epidermidis*	70 kHz 3 W/cm^2^ CW, 1, 2, 3, 6 h	Gentamicin Streptomycin Kanamycin Tetracycline Ampicillin	Simultaneous application of ultrasound and antibiotic significantly increased the effectiveness of the selected antibiotics. Bacterial viability was reduced by several orders of magnitude when harmless levels of ultrasound were combined with the selected antibiotics, especially the aminoglycosides.

Rediske et al. (1999) [18]	*In vitro* study on planktonic cultures	*P. aeruginosa*	70 kHz 2.2 W/cm^2^ CW, 1, 3, 6, 12 h	Erythromycin	Ultrasound in combination with erythromycin reduced the viability of *P. aeruginosa* by 1-2 orders of magnitude compared with antibiotic alone, even at concentrations below MIC.

Rapoport et al (1999) [17]	*In vitro* study on planktonic cultures	*P. aeruginosa* *E. coli*	80 kHz 0.8–2.4 W/cm^2^ CW, 2 h	Gentamicin	The penetration of spin-labeled gentamicin was not affected by insonation below the cavitation threshold. It implies that synergistic effect between hydrophilic antibiotics and LFU in killing Gram-negative bacteria did not result from the enhanced antibiotic penetration through bacterial cell walls.

Runyan et al. (2006) [20]	*In vitro* study on planktonic cultures	*P. aeruginosa*	70 kHz 0.5–4.7 W/cm^2^ CW, 3 min	Nitrocefin	The rate of nitrocefin hydrolysis is increased by ultrasound in an intensity-dependent manner, which reflects the rate of entry of the antibiotic.

Ayan et al. (2008) [23]	*In vitro* study on planktonic cultures	*S. aureus*	1.5 MHz 0.03–0.161 W/cm^2^ CW, 20 min	Penicillin Oxacillin Teicoplanin Vancomycin Erythromycin Clindamycin Levofloxacin Ciprofloxacin	The samples treated with LFU showed a significantly lower number of bacteria colonies compared to the antibiotic alone. Partial destruction or disintegration of the cell walls was detected in some bacteria using the electron microscopy.

Conner-Kerr et al. (2010) [24]	*In vitro* study on planktonic cultures	MRSA	35 kHz 2.0 W/cm^2^ CW, 0.5, 1, 3 min	Oxacillin	LFU reduces CFU of bacteria, punctures, and fractures cell walls and alters colonial characteristics of MRSA, including resistance to the methicillin.

Liu et al. (2011) [21]	*In vitro* study on planktonic cultures	*E. coli*	40 kHz 1 W/cm^2^ CW, 15, 30, 45 min	Levofloxacin Ciprofloxacin	Addition of LFU to levofloxacin and ciprofloxacin exposure enhances the effectiveness of the antibiotics in killing *E. coli*. LFU can activate fluoroquinolones to produce reactive oxygen species, which are mainly determined as superoxide radical anion and hydroxyl radical.

Zhu et al. (2014) [16]	*In vitro* study on planktonic cultures	*E. coli*	46.5 kHz 100 mW/cm^2^ 1 : 3 duty cycle	Gentamicin	Microbubble-mediated LFU could further enhance the antimicrobial efficacy of gentamicin compared with LFU alone. Transmission electron microscopy images showed more destruction and higher thickness of bacterial cell membranes in the microbubble-mediated LFU than those in other groups.

CW: continuous wave ultrasound.

**Table 2:** Reports about effect of antibiotics combined with LFU on biofilm.

Authors (year)	Type of research	Pathogens	Frequency, density, and time of LFU	Combined antibiotics	Results
Qian et al. (1996) [26]	*In vitro *study on biofilm	*P. aeruginosa*	500 kHz Average insonation intensity of 10 mW/cm^2^ CW, 2 h	Gentamicin	LFU enhanced bacteria bactericidal effect of gentamicin on 24 h old biofilm of *P. aeruginosa.* CLSM results showed that LFU does not disrupt biofilm or disperse the bacteria.

Qian et al. (1997) [27]	*In vitro *study on biofilm	*P. aeruginosa*	70, 500 kHz 2.25, 10 MHz 10 mW/cm^2^ CW	Gentamicin	A significantly greater fraction of the bacteria was killed by gentamicin when they were subjected to ultrasound. Ultrasound by itself did not have any deleterious effect on the biofilm viability. LFU is significantly more effective than higher frequency ultrasound in reducing bacterial viability within the biofilm.

Johnson et al. (1998) [29]	*In vitro *study on biofilm	*E. coli*	70, 500 kHz 20, 100 mW/cm^2^ CW, 2, 4, 6 h	Gentamicin	The combination of 70 kHz ultrasound with the antibiotic more significantly reduced bacterial viability than antibiotic alone, resulting in about 97% killing in 2 h. The combination of 500 kHz ultrasound and antibiotic produced only a slight, insignificant reduction in the killing caused by antibiotic alone.

Rediske et al. (1999) [30]	*In vivo* study of biofilm-infected rabbit model	*E. coli*	28.48 kHz 100–300 mW/cm^2^ CW, 24 h	Gentamicin	Exposure to ultrasound only caused no significant difference in bacterial viability. But in the presence of antibiotic, bacterial viability was significantly reduced due to 300 mW/cm^2^ ultrasound (*P* = 0.0485) and insignificant reduction due to 100 mW/cm^2^ ultrasound.

Qian et al. (1999) [28]	*In vitro *study on biofilm	*P. aeruginosa*	44 kHz–10 MHz 1 and 10 mW/cm^2^ duty cycle of 1 : 10 2 h	Gentamicin	The enhanced bactericidal effect of antibiotic due to sonication showed a monotonic decrease as the frequency increased from 44 kHz to 10 MHz, indicating that the lower frequencies are more effective in enhancing the antibiotic action. A power density of 10 mW/cm^2^ is more effective in enhancing the bactericidal effect of the antibiotic than the 1 mW/cm^2^ intensity.

Rediske et al. (2000) [31]	*In vivo* study of biofilm-infected rabbit model	*E. coli*	28.48 kHz, 1 : 3 duty cycle with 300 mW/cm^2^ 1 : 6 duty cycle with 600 mW/cm^2^ 24 h	Gentamicin	The average bacterial viability was reduced from 2.94 to 0.99 log⁡10 CFU/cm^2^ by 300 mW/cm^2^ pulsed ultrasound and from 2.93 to 1.69 log⁡10 CFU/cm^2^ by 600 mW/cm^2^ pulsed ultrasound. No discoloration or damage of the skin was apparent.

Carmen et al. (2004) [34]	*In vivo* study of biofilm-infected rabbit model	*S. epidermidis*	28.48 kHz 1 : 3 duty cycle 500 mW/cm^2^ 24, 48 h	Vancomycin	Application of LFU enhanced the activity of vancomycin against implanted *S. epidermidis* biofilms. 48 h of insonation significantly reduced the count of viable bacteria in the biofilm.

Carmen et al. (2004) [32]	*In vitro *study on biofilm	*P. aeruginosa* *E. coli*	70-kHz 1.9 and 2.9 W/cm^2^ for *E. coli* 1.5 and 2.5 W/cm^2^ for *P. aeruginosa* CW, 15, 30, 45 min	Gentamicin	Ultrasonication significantly increased transport of gentamicin across biofilms that normally blocked or slowed gentamicin transport when not exposed to ultrasound.

Carmen et al. (2005) [33]	*In vivo* study of biofilm-infected rabbit model	*P. aeruginosa* *E. coli*	28.5 kHz 1 : 3 duty cycle 500 mW/cm^2^ 24, 48 h	Gentamicin	The number of viable bacteria in *E. coli* biofilm was reduced to 2.29 ± 0.40 log⁡10 CFU/cm^2^ after 72 h of treatment with gentamicin alone and to 0.011 ± 1.02 log⁡10 CFU/cm^2^ after treatment with both gentamicin (for 72 h) and ultrasound (for 48 h) compared with that after treatment with antibiotic alone. But 24 or 48 h of ultrasound combined with gentamicin failed to significantly enhance the bactericidal effect of P. aeruginosa in the biofilm.

Seth et al. (2013) [35]	*In vivo* study of biofilm-infected rabbit model	*P. aeruginosa*	3 min every other day or every day as instructions of MIST Therapy System	Ciprofloxacin (topical)	LFU has a significant impact on biofilm-infected wounds, including a decrease in viable bacteria and an overall improvement in wound healing and host inflammatory dynamics.

Li et al. (2015) [36]	*In vivo* study of biofilm-infected mouse model	*S. epidermidis* *S. aureus*	200 mW/cm^2^ 1 : 1 duty cycle 20 min, 3 times a day	HBD-3	Biofilm densities, the percentage of live cells, and the viable counts from the biofilm on the titanium surface in mice were significantly decreased in the group of the HBD-3 combined with ultrasound targeted microbubble destruction.

Liu et al. (2016) [37]	*In vitro *study on biofilm	Pan-resistant *A. baumannii*	40 kHz 1 : 9 duty cycle 600 mW/cm^2^ 30 min	Colistin Vancomycin	Reductions > 2 log CFU/mL were observed for colistin plus vancomycin with LFU than without LFU after 12 h of incubation. Bacterial counts declined continuously for 24 h, with a reduction of 3.77 log CFU/mL from with LFU to without LFU.

CW: continuous wave ultrasound.

**Table 3:** Reports about effect of LFU on antibiotic release from implanted material.

Authors (year)	Type of research	Pathogens	Frequency, density, and time of LFU	Loaded antibiotics	Results
Hendriks et al. (2003) [40]	*In vitro* study of bone cement	NA	46.5 kHz Time average acoustic intensity of 167 mW/cm^2^ 1 : 3 duty cycle 18 h	Gentamicin	Average gentamicin release was higher in the insonated samples for all three bone cements, although none of these differences were statistically significant.

Norris et al. (2005) [39]	*In vitro* studyon hydrogel polymer matrix	*P. aeruginosa*	43 kHz CW, 15 min at three time points in 4 h period	Ciprofloxacin	Biofilm accumulation on ciprofloxacin-loaded hydrogels with ultrasound-induced drug delivery was significantly reduced compared to the accumulation of biofilms grown in control experiments.

Ensing et al. (2005) [41]	*In vitro* studyon bone cement samples and beads	NA	46.5 kHz Average acoustic intensity of 167 mW/cm^2^ 1 : 3 duty cycle 18 h	Gentamicin	Pulsed ultrasound significantly enhanced gentamicin release from gentamicin-loaded beads, whereas gentamicin release from the gentamicin-loaded bone cements was not significantly enhanced.

Ensing et al. (2005) [43]	*In vivo* study of bone cement	*E. coli*	28.48 kHz Mean acoustic intensity of 167 mW/cm^2^ 1 : 3 duty cycle 24–72 h	Gentamicin	Ultrasound in combination with gentamicin yielded a tendency towards enhanced bacterial killing in biofilms growing on acrylic bone cements.

Ensing et al. (2006) [42]	*In vitro* study	*E. coli* *S. aureus*Coagulase-negative staphylococci *P. aeruginosa*	46.5 kHz mean acoustic intensity of 167 mW/cm^2^ 1 : 3 duty cycle 40 h	Gentamicin Gentamicin/clindamycin	Application of pulsed ultrasound in combination with antibiotic release by antibiotic-loaded bone cements yielded a reduction of both planktonic and biofilm bacterial viability compared with antibiotic release without application of ultrasound.

Cai et al. (2007) [44]	*In vivo* study of bone cement	*S. aureus*	46.5 kHz Average intensity of 300 mW/cm^2^ 2 : 1 duty cycle 12 h	Vancomycin	The enhanced reduction in hip aspirates from ultrasound group 0–12 h was 1.62 log⁡10 CFU/ml (*P* < 0.05), and that in hip aspirates from ultrasound group 12–24 was 2.77 log⁡10 CFU/ml (*P* < 0.01).

Yan et al. (2007) [46]	*In vitro* and* in vivo *study of antibiotic-loaded bone cement	*S. aureus*	25 kHz 100 or 300 mW/cm^2^ for *in vitro* study 300 mW/cm^2^ for *in vivo* study CW, 0.5 h	Vancomycin	LFU increased the drug elution by 2.57–27.44% when compared with the controls *in vitro*. LFU increased local *T*_max_ by 47.6 mg/mL and urinary elimination of vancomycin by 109.56 mg but failed to prolong local *T* > MIC. Decreased bacterial vitality and relieved inflammation in the infected hip treated were also observed in ultrasound group.

Cai et al. (2009) [10]	*In vitro* study of bone cement	NA	46.5 kHz Spatial-average-time-average intensity of 1.2 W/cm^2^ 1 : 2 duty cycle 40 min	Vancomycin	Intermittent watt-level ultrasonication improved the ultrasound-enhanced vancomycin release from cement in view of the prolonged *T* > MIC and the inhibited subtherapeutic release compared with continuous ultrasonication.

Lin et al. (2015) [45]	*In vitro* and* in vivo *study of PMMA cement	*S. aureus*	1 MHz 300 mW/cm^2^ 3 : 10 duty cycle 4–24 h	Vancomycin	*In vitro* study showed the eluted vancomycin from the vancomycin + ultrasound + microbubbles group was significantly more effective against the planktonic *S. aureus* than in any of the other study groups (*P* < 0.001). *In vivo *study showed the viable count of *S. aureus* that survived when vancomycin + ultrasound + microbubbles cylinders were implanted was significantly lower than in each of the other study groups (*P* < 0.0001).

Wendling et al. (2016) [47]	*In vitro* study of PMMA cement	NA	25.5 kHz CW, 5, 15, 45 min	Vancomycin	There were significant increases in elution amount for LFU treatment groups compared with the non-LFU groups (*P* < 0.03). The correlations between LFU duration and total elution amount were significant (*P* = 0.03).

CW: continuous wave ultrasound. NA: not applicable.

## References

[1] Golkar Z., Bagasra O., Pace D. G. (2014). Bacteriophage therapy: A potential solution for the antibiotic resistance crisis. *Journal of Infection in Developing Countries*.

[2] Ventola C. L. (2015). The antibiotic resistance crisis—part 1: causes and threats. *Pharmacy and Therapeutics*.

[3] Doi Y., Murray G. L., Peleg A. Y. (2015). Acinetobacter baumannii: evolution of antimicrobial resistance-treatment options. Semin Respir Crit Care Med. *Seminars in Respiratory and Critical Care Medicine*.

[4] Sun Y., Cai Y., Liu X., Bai N., Liang B., Wang R. (2013). The emergence of clinical resistance to tigecycline. *International Journal of Antimicrobial Agents*.

[5] Cai Y., Chai D., Wang R., Liang B., Bai N. (2012). Colistin resistance of Acinetobacter baumannii: clinical reports, mechanisms and antimicrobial strategies. *Journal of Antimicrobial Chemotherapy*.

[6] Cai Y., Bai N., Liu X. (2016). Alone or in combination?. *Infectious Diseases (Lond)*.

[7] Yu H., Chen S., Cao P. (2012). Synergistic bactericidal effects and mechanisms of low intensity ultrasound and antibiotics against bacteria: A review. *Ultrasonics Sonochemistry*.

[8] Gao S., Lewis G. D., Ashokkumar M., Hemar Y. (2014). Inactivation of microorganisms by low-frequency high-power ultrasound: 1. Effect of growth phase and capsule properties of the bacteria. *Ultrasonics Sonochemistry*.

[9] Ashokkumar M. (2011). The characterization of acoustic cavitation bubbles—An overview. *Ultrasonics Sonochemistry*.

[10] Cai X. Z., Chen X. Z., Yan S. G., Ruan Z. R., Yan R. J., Ji K. (2009). Intermittent watt-level ultrasonication facilitates vancomycin release from therapeutic acrylic bone cement. *Journal of Biomedical Materials Research: Applied Biomaterials*.

[11] Al Bsoul A., Magnin J.-P., Commenges-Bernole N., Gondrexon N., Willison J., Petrier C. (2010). Effectiveness of ultrasound for the destruction of Mycobacterium sp. strain (6PY1). *Ultrasonics Sonochemistry*.

[12] Serena T., Lee S. K., Lam K., Attar P., Meneses P., Ennis W. (2009). The impact of noncontact, nonthermal, low-frequency ultrasound on bacterial counts in experimental and chronic wounds. *Ostomy Wound Management*.

[13] Singer A. J., Coby C. T., Singer A. H., Thode Jr. H. C., Tortora G. T. (1999). The effects of low-frequency ultrasound on Staphylococcus epidermidis. *Current Microbiology*.

[14] Pitt W. G., McBride M. O., Lunceford J. K., Roper R. J., Sagers R. D. (1994). Ultrasonic enhancement of antibiotic action on gram-negative bacteria. *Antimicrobial Agents and Chemotherapy*.

[15] Williams R. G., Pitt W. G. (1997). In vitro response of Escherichia coli to antibiotics and ultrasound at various insonation intensities. *Journal of Biomaterials Applications*.

[16] Zhu H. X., Cai X. Z., Shi Z. L., Hu B., Yan S. G. (2014). Microbubble-mediated ultrasound enhances the lethal effect of gentamicin on planktonic Escherichia coli. *BioMed Research International*.

[17] Rapoport N., Smirnov A. I., Pitt W. G., Timoshin A. A. (1999). Bioreduction of Tempone and spin-labeled gentamicin by gram-negative bacteria: Kinetics and effect of ultrasound. *Archives of Biochemistry and Biophysics*.

[18] Rediske A. M., Rapoport N., Pitt W. G. (1999). Reducing bacterial resistance to antibiotics with ultrasound. *Letters in Applied Microbiology*.

[19] Rapoport N., Smirnov A. I., Timoshin A., Pratt A. M., Pitt W. G. (1997). Factors affecting the permeability of Pseudomonas aeruginosa cell walls toward lipophilic compounds: Effects of ultrasound and cell age. *Archives of Biochemistry and Biophysics*.

[20] Runyan C. M., Carmen J. C., Beckstead B. L., Nelson J. L., Robison R. A., Pitt W. G. (2006). Low-frequency ultrasound increases outer membrane permeability of *Pseudomonas aeruginosa*. *Journal of General and Applied Microbiology*.

[21] Liu B., Wang D. J., Liu B. M., Wang X., LL He., Wang J. (2011). The influence of ultrasound on the fluoroquinolones antibacterial activity. *Ultrason Sonochem*.

[22] Rediske A. M., Hymas W. C., Wilkinson R., Pitt W. G. (1998). Ultrasonic enhancement of antibiotic action on several species of bacteria. *Journal of General and Applied Microbiology*.

[23] Ayan I., Aslan G., Comelekoglu U., Yilmaz N., Colak M. (2008). The effect of low-intensity pulsed sound waves delivered by the Exogen device on Staphylococcus aureus morphology and genetics. *Acta Orthopaedica et Traumatologica Turcica*.

[24] Conner-Kerr T., Alston G., Stovall A. (2010). The effects of low-frequency ultrasound (35 kHz) on methicillin-resistant staphylococcus aureus (MRSA) in vitro. *Ostomy Wound Management*.

[25] Malone M., Goeres D. M., Gosbell I., Vickery K., Jensen S., Stoodley P. (2017). Approaches to biofilm-associated infections: the need for standardized and relevant biofilm methods for clinical applications. *Expert Review of Anti-Infective Therapy*.

[26] Qian Z., Stoodley P., Pitt W. G. (1996). Effect of low-intensity ultrasound upon biofilm structure from confocal scanning laser microscopy observation. *Biomaterials*.

[27] Qian Z., Sagers R. D., Pitt W. G. (1997). The effect of ultrasonic frequency upon enhanced killing of P. aeruginosa biofilms. *Annals of Biomedical Engineering*.

[28] Qian Z., Sagers R. D., Pitt W. G. (1999). Investigation of the mechanism of the bioacoustic effect. *Journal of Biomedical Materials Research*.

[29] Johnson L. L., Peterson R. V., Pitt W. G. (1998). Treatment of bacterial biofilms on polymeric biomaterials using antibiotics and ultrasound. *Journal of Biomaterials Science*.

[30] Rediske A. M., Roeder B. L., Brown M. K., Nelson J. L., Robison R. L., Draper D. O. (1999). Ultrasonic enhancement of antibiotic action on Escherichia coli biofilms: an in vivo model. *Antimicrob Agents Chemother*.

[31] Rediske A. M., Roeder B. L., Nelson J. L. (2000). Pulsed ultrasound enhances the killing of Escherichia coli biofilms by aminoglycoside antibiotics in vivo. *Antimicrobial Agents and Chemotherapy*.

[32] Carmen J. C., Nelson J. L., Beckstead B. L. (2004). Ultrasonic-enhanced gentamicin transport through colony biofilms of Pseudomonas aeruginosa and Escherichia coli. *Journal of Infection and Chemotherapy*.

[33] Carmen J. C., Roeder B. L., Nelson J. L. (2005). Treatment of biofilm infections on implants with low-frequency ultrasound and antibiotics. *The American Journal of Infection Control*.

[34] Carmen J. C., Roeder B. L., Nelson J. L., Beckstead B. L., Runyan C. M., Schaalje G. B. (2004). Ultrasonically enhanced vancomycin activity against Staphylococcus epidermidis biofilms in vivo. *Journal of Biomaterials Applications*.

[35] Seth A. K., Nguyen K. T., Geringer M. R. (2013). Noncontact, low-frequency ultrasound as an effective therapy against *Pseudomonas aeruginosa*-infected biofilm wounds. *Wound Repair and Regeneration*.

[36] Li S., Zhu C., Fang S., Zhang W., He N., Xu W. (2015). Ultrasound microbubbles enhance human beta-defensin 3 against biofilms. *Journal of Surgical Research*.

[37] Liu X., Yin H., Weng C.-X., Cai Y. (2016). Low-Frequency Ultrasound Enhances Antimicrobial Activity of Colistin-Vancomycin Combination against Pan-Resistant Biofilm of Acinetobacter baumannii. *Ultrasound in Medicine and Biology*.

[38] Mihai M. M., Holban A. M., Giurcaneanu C., Popa L. G., Oanea R. M., Lazar V. (2015). Microbial biofilms: impact on the pathogenesis of periodontitis, cystic fibrosis, chronic wounds and medical device-related infections. *Current Topics in Medicinal Chemistry*.

[39] Norris P., Noble M., Francolini I. (2005). Ultrasonically controlled release of ciprofloxacin from self-assembled coatings on poly(2-hydroxyethyl methacrylate) hydrogels for Pseudomonas aeruginosa biofilm prevention. *Antimicrobial Agents and Chemotherapy*.

[40] Hendriks J. G. E., Ensing G. T., Van Horn J. R., Lubbers J., Van Der Mei H. C., Busscher H. J. (2003). Increased release of gentamicin from acrylic bone cements under influence of low-frequency ultrasound. *Journal of Controlled Release*.

[41] Ensing G. T., Hendriks J. G., Jongsma J. E., van Horn J. R., van der Mei H. C., Busscher H. J. (2005). The influence of ultrasound on the release of gentamicin from antibiotic-loaded acrylic beads and bone cements. *Journal of Biomedical Materials Research*.

[42] Ensing G. T., Neut D., van Horn J. R., van der Mei H. C., Busscher H. J. (2006). The combination of ultrasound with antibiotics released from bone cement decreases the viability of planktonic and biofilm bacteria: an in vitro study with clinical strains. *Journal of Antimicrobial Chemotherapy*.

[43] Ensing G. T., Roeder B. L., Nelson J. L., van Horn J. R., van der Mei H. C., Busscher H. J. (2005). Effect of pulsed ultrasound in combination with gentamicin on bacterial viability in biofilms on bone cements in vivo. *Journal of Applied Microbiology*.

[44] Cai X. Z., Yan S. G., Wu H. ., He R. X., Dai X. S., Chen H. X. (2007). Effect of delayed pulsed-wave ultrasound on local pharmacokinetics and pharmacodynamics of vancomycin-loaded acrylic bone cement in vivo. *Antimicrobial Agents and Chemotherapy*.

[45] Lin T., Cai X. Z., Shi M. M., Ying Z. M., Hu B., Zhou C. H. (2015). In vitro and in vivo evaluation of vancomycin-loaded PMMA cement in combination with ultrasound and microbubbles-mediated ultrasound. *BioMed Research International*.

[46] Yan S., Cai X., Yan W., Dai X., Wu H. (2007). Continuous wave ultrasound enhances vancomycin release and antimicrobial efficacy of antibiotic-loaded acrylic bone cement in vitro and in vivo. *Journal of Biomedical Materials Research Applied Biomater*.

[47] Wendling A., Mar D., Wischmeier N., Anderson D., McIff T. (2016). Combination of modified mixing technique and low frequency ultrasound to control the elution profile of vancomycin-loaded acrylic bone cement. *Bone and Joint Research*.

[48] Breuing K. H., Bayer L., Neuwalder J., Orgill D. P. (2005). Early experience using low-frequency ultrasound in chronic wounds. *Annals of Plastic Surgery*.

[49] Tewarie L., Moza A. K., Zayat R., Autschbach R., Goetzenich A., Menon A. K. (2014). Ultrasound-assisted treatment of sternocutaneous fistula in post-sternotomy cardiac surgery patients. *European Journal of Cardio-thoracic Surgery*.

[50] Komrakov V. E., Antipov S. V. (1990). Use of ultrasonics and antibiotics in the treatment of wounds in patients with high risk of infection of vascular transplants. *Klinicheskaia khirurgiia*.

[51] Zhao Q. H., Zhu F. B., Cai X. Z., Yan S. G., He R. X. (2017). Effects of low-frequency pulsed wave ultrasound on the shear properties of the interface of vancomycin-loaded acrylic bone cement-stem. *Zhonghua Yi Xue Za Zhi*.

[52] Michailidis L., Kotsanas D., Orr E. (2016). Does the new low-frequency ultrasonic debridement technology pose an infection control risk for clinicians, patients, and the clinic environment?. *American Journal of Infection Control*.

